# Laccase Affects the Rate of Cryptococcus neoformans Nonlytic Exocytosis from Macrophages

**DOI:** 10.1128/mBio.02085-20

**Published:** 2020-09-08

**Authors:** Stefânia de Oliveira Frazão, Herdson Renney de Sousa, Lenise Gonçalves da Silva, Jéssica dos Santos Folha, Kaio César de Melo Gorgonha, Getúlio Pereira de Oliveira, Maria Sueli Soares Felipe, Ildinete Silva-Pereira, Arturo Casadevall, André Moraes Nicola, Patrícia Albuquerque

**Affiliations:** a Laboratory of Molecular Biology of Pathogenic Fungi, Laboratory of Molecular Biology, Department of Cell Biology, Institute of Biological Sciences, University of Brasília, Brasília, Brazil; b Microbiology, Immunology, and Biotechnology Laboratory, Faculty of Medicine, University of Brasília, Brasília, Brazil; c Division of Allergy and Inflammation, Department of Medicine, Beth Israel Deaconess Medical Center, Harvard Medical School, Boston, Massachusetts, USA; d Graduate Program in Genomic Sciences and Biotechnology, Catholic University of Brasília, Brasília, Brazil; e Department of Molecular Microbiology and Immunology, Johns Hopkins Bloomberg School of Public Health, Baltimore, Maryland, USA; f Faculty of Ceilândia, University of Brasília, Brasília, Brazil; Institut Pasteur

**Keywords:** *Cryptococcus neoformans*, laccase, macrophages, nonlytic exocytosis

## Abstract

Nonlytic exocytosis is a process in which previously ingested microbes are expelled from host phagocytes with the concomitant survival of both cell types. This process has been observed in the interaction of *Cryptococcus* spp. and other fungal cells with phagocytes as distant as mammalian, bird, and fish macrophages and ameboid predators. Despite a great amount of research dedicated to unraveling this process, there are still many questions about its regulation and its final benefits for host or fungal cells. During a study to characterize the virulence attributes of Brazilian clinical isolates of C. neoformans, we observed great variability in their rates of nonlytic exocytosis and noted a correlation between this process and fungal melanin production/laccase activity. Flow cytometry experiments using melanized cells, nonmelanized cells, and *lac1*Δ mutants revealed that laccase has a role in the process of nonlytic exocytosis that seems to be independent of melanin production. These results identify a role for laccase in virulence, independent of its role in pigment production, that represents a new variable in the regulation of nonlytic exocytosis.

## Abstract (precis)

Cryptococcus neoformans is a yeast that causes severe disease, primarily in immunosuppressed people. It has many attributes that allow it to survive and cause disease, such as a polysaccharide capsule and the dark pigment melanin produced by the laccase enzyme. Upon infection, the yeast is ingested by cells called macrophages, whose function is to kill them. Instead, these fungal cells can exit from macrophages in a process called nonlytic exocytosis. We know that this process is controlled by both host and fungal factors, only some of which are known. As part of an ongoing study, we observed that C. neoformans isolates that produce melanin faster are more-frequent targets of nonlytic exocytosis. Further experiments showed that this is probably due to higher production of laccase, because fungi lacking this enzyme are nonlytically exocytosed less often. This shows that laccase is an important signal/regulator of nonlytic exocytosis of C. neoformans from macrophages.

## OBSERVATION

Cryptococcus neoformans is an encapsulated yeast that causes cryptococcosis, a globally distributed disease whose most frequent manifestation is a severe meningoencephalitis in immunosuppressed people. Infection normally occurs by inhalation, which leads to fungal killing or containment within granulomas in immunocompetent hosts. Macrophages are considered the main effector cells in the immune response against C. neoformans but also provide a site for intracellular replication and a vehicle for systemic dispersion of this fungus. The interaction of C. neoformans cells with macrophages after phagocytosis can lead to a number of outcomes, both for the fungi (death, latency, intracellular proliferation) and for the phagocyte (cell lysis, cell division, transfer of the phagocytosed cargo to another macrophage) (1). An additional outcome is nonlytic exocytosis, also called vomocytosis, a process in which previously ingested microbes are expelled from host cells without compromising the viability of either (2, 3). Several studies have provided evidence that both fungal and host factors can influence nonlytic exocytosis. For C. neoformans, these include fungal viability, the acid-base properties of the capsule, or urease activity inhibiting macrophage phagosome maturation. On the host side, immunity status, macrophage polarization by different cytokines, and viral coinfections are known to affect this process (2, 4–7).

For a few years, our team has been phenotypically characterizing virulence-related features of C. neoformans VNI clinical isolates obtained from patients with cryptococcosis studied by the Cryptococcosis Brazil Network. Among several different features, we have been analyzing the rates of nonlytic exocytosis of the different isolates, their phagocytosis and survival after interaction with macrophages, and their melanization. Nonlytic exocytosis was analyzed using time-lapse microscopy as described previously with some modifications (8). Fungal phagocytosis and survival were analyzed after 2 or 24 h of interaction with BALB/c bone marrow-derived macrophages (BMDM), respectively, as described previously (9, 10). Experiments with mice were approved by the Animal Ethics Committee of the University of Brasilia (UnB DOC 52657/2011). Melanization kinetics was assessed by spotting cultures of each isolate in l-3,4-dihydroxyphenylalanine (l-DOPA) minimal medium and performing densitometry on images collected every 12 h (see Fig. S1 in the supplemental material). We also established a subjective melanization score for each isolate based on how fast its melanization happened and how much darker the colony was at the end of the analysis; both methodologies are described in detail elsewhere (11).

10.1128/mBio.02085-20.1 FIG S1 Methods for the assessment of melanization among the clinical isolates. (A) Melanization kinetics of the different isolates based on densitometry of images of colonies of each isolate collected every 12 h. Shown is the nonlinear regression curve of median gray values at each observation time (representing the amount of melanin measured). This regression resulted in the melanization parameter t_HMM_, the time it takes for the colony to reach half of its final melanization intensity. (B) Representative photos of the macroscopic melanization profiles of four clinical isolates and the standard H99 internal-control strain in all experiments. Experiments were repeated at least twice with similar results. Download FIG S1, TIF file, 1.9 MB. Copyright © 2020 Frazão et al. 2020 Frazão et al. This content is distributed under the terms of the Creative Commons Attribution 4.0 International license.

**FIG 1:**
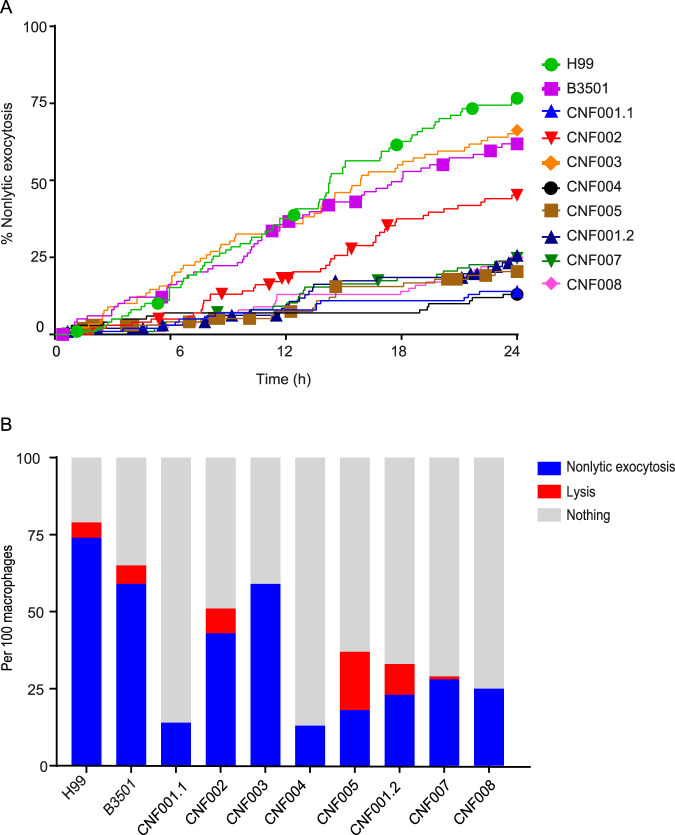
Outcomes of macrophage interaction with different C. neoformans isolates. (A) Kinetics of events of macrophages undergoing nonlytic exocytosis or macrophage lysis after interaction with different C. neoformans isolates for 24 h. Kaplan-Meier curves were generated to follow the macrophages after the interaction with each isolate. Nonlytic exocytosis events are represented by stepwise changes in the curves, whereas each corresponding symbol (circle, square, triangle, etc.) along the curves represents a macrophage lysis event. (B) Outcome of macrophages with internalized C. neoformans after 24 h. One hundred macrophages were followed for each isolate, and each bar displays the proportions of events of nonlytic exocytosis (blue), macrophage lysis (red), or no observed event (gray).

The main strategy of our ongoing study involved evaluating virulence and host-pathogen attributes in different strains and correlating these data with clinical outcomes in order to understand their role in human disease. We have not yet measured nonlytic exocytosis in a sufficient number of isolates to draw clinically relevant conclusions, but the experiments with the first batch of clinical isolates led to several interesting observations that could not have been made with the limited number of laboratory-adapted fungal strains used in previous studies. Macrophages underwent nonlytic exocytosis beginning around 2 h after the start of image collection and continuing during the 24 h of observation for all isolates (Fig. 1A). The rates ranged from 14% to 74%, and all the clinical isolates presented lower rates of nonlytic exocytosis than the control strain, H99 (Fig. 1B). Lytic events were also observed in all the interactions but were considerably less frequent than nonlytic exocytosis.

**FIG 2:**
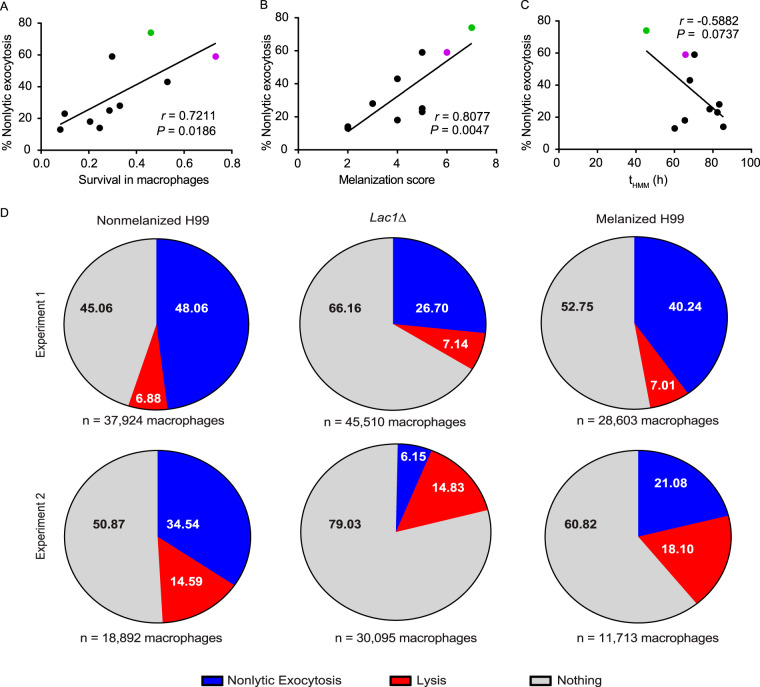
Correlations and hypothesis testing. (A) Correlation between the percentage of macrophages undergoing nonlytic exocytosis and the survival of different C. neoformans isolates after interaction with BMDM for 24 h. (B) Correlation between the percentage of macrophages undergoing nonlytic exocytosis and the melanization score for each C. neoformans isolate. (C) Correlation between the percentage of macrophages undergoing nonlytic exocytosis and the time for half-maximum melanization (t_HMM_) for each isolate. Pearson’s correlation coefficient (*r*) and *P* values are shown in each separate graph. Each point corresponds to one C. neoformans isolate, and the points corresponding to control strains H99 and B3501 are colored green and purple, respectively. The solid lines exhibit the best lines fitted to the data based on a simple regression model. (D) Percentages of macrophages that underwent nonlytic exocytosis and lysis after the interaction of macrophages with nonmelanized H99, melanized H99, and a *lac1*Δ mutant as determined by two independent flow cytometry experiments, performed on different days.

We correlated the percentage of nonlytic exocytosis events after macrophage interaction with the different isolates with several virulence phenotypes. We found no significant correlation with the basal capsule size in Sabouraud medium (Fig. S2A) or the isolate’s ability to modulate capsule size after growth in minimal medium (Fig. S2B) or Sabouraud-morpholinepropanesulfonic acid (MOPS) (Fig. S2C). However, we did observe interesting results with survival rates for each isolate and their melanization kinetics and scores. We observed a positive correlation (*r* = 0.7211; *P = *0.0186) between the rate of nonlytic exocytosis and an isolate’s survival after interaction with macrophages (Fig. 2A) but no correlation between nonlytic exocytosis and the percentage of phagocytosis (Fig. S2D). This suggests a possible link between the ability of the fungus to survive/grow within the macrophages and its ability to trigger nonlytic exocytosis, in agreement with previous work showing that fungal proliferation rates can affect nonlytic exocytosis (3, 7).

10.1128/mBio.02085-20.2 FIG S2 Correlation between nonlytic exocytosis and the isolate’s capsule size under different conditions or the percentage of phagocytosis by macrophages. (A) Correlation between nonlytic exocytosis and the capsule size of the isolates under noninducing conditions (Sabouraud medium). (B) Correlation between nonlytic exocytosis and the average percentage of increase in the capsule size of the isolates after growth in minimal medium (MM). (C) Correlation between nonlytic exocytosis and the average percentage of increase in the capsule size of the isolates after growth in Sabouraud-MOPS medium. (D) Correlation between nonlytic exocytosis and the percentages of phagocytosis of the different isolates by macrophages. Pearson’s correlation coefficient (*r*) and *P* values are shown in each separate graph. Each point corresponds to one C. neoformans isolate, and the points corresponding to control strains H99 and B3501 are depicted in green and purple, respectively. The solid line exhibits the best line fitted to the data based on a simple regression model. Download FIG S2, EPS file, 0.8 MB. Copyright © 2020 Frazão et al. 2020 Frazão et al. This content is distributed under the terms of the Creative Commons Attribution 4.0 International license.

Another interesting correlation was observed between nonlytic exocytosis and each isolate’s melanization score. Those with the highest melanization scores also manifested the highest ability to escape the macrophage by nonlytic exocytosis (*r* = 0.8077; *P = *0.0047) (Fig. 2B). A similar correlation was observed regarding the kinetics of melanization: isolates that melanized faster (presenting a lower time for half-maximum melanization [t_HMM_]) also manifested the highest rates of nonlytic exocytosis (*r* = –0.5882; *P = *0.0737) (Fig. 2C).

These correlations suggested that melanin and/or laccase could be involved in nonlytic exocytosis. To test these hypotheses, we performed two flow cytometry experiments using a modification of a previously described protocol (12): a gain-of-function assay with premelanized cells, in which we should observe more nonlytic exocytosis if melanin is involved, and a loss-of-function assay, in which a laccase knockout mutant would be exocytosed less often if laccase played a role in this process. Briefly, DDAO-SE [9-*H*-(1,3-dichloro-9,9-dimethylacridin-2-one-7-yl)-succinimidyl ester]-labeled J774.16 macrophages were incubated for 2 h with fungal cells previously labeled with CMFDA (5-chloromethylfluorescein diacetate). After the interaction, extracellular yeasts were stained with Uvitex 2B, a fungal cell wall dye that is unable to permeate intact macrophage membranes. Following that, macrophages containing ingested C. neoformans were selected using fluorescence-activated cell sorter gating for events positive for DDAO-SE and CMFDA but negative for Uvitex 2B (for the gating strategy, see Fig. S3). Those cells were plated and 24 h later were analyzed by flow cytometry in the presence of DAPI (4′,6-diamidino-2-phenylindole) to evaluate if the macrophages had undergone lysis or were still alive but no longer harbored C. neoformans cells, corresponding to macrophages that had undergone nonlytic exocytosis (DDAO-SE positive, CMFDA negative). As shown in Fig. 2D, there was little variation among the macrophage lytic events among the three different samples (red regions in the pie charts). In the gain-of-function assay, we actually observed a decrease in nonlytic exocytosis of premelanized cells in comparison to nonmelanized cells in both experiments, from 48.06% to 40.24% (*P* < 0.0001 by the chi-square test) and from 34.54% to 21.08% (*P* < 0.0001 by the chi-square test), respectively. On the other hand, the loss-of-function assay showed a decrease in the proportion of nonlytic exocytosis events in both experiments, from 48.06% to 26.7% (*P* < 0.0001 by the chi-square test) and from 34.54% to 6.15% (*P* < 0.0001 by the chi-square test), respectively (Fig. 2D). These results suggest that laccase itself, rather than melanization, has a major role in the process of nonlytic exocytosis. This dissociation between melanization and laccase is not surprising given that the enzyme mediates other functions apart from pigment production, including affecting the oxidative burst, lipid oxidation, and prostaglandin synthesis (13, 14). Furthermore, these results establish a role for laccase independently of any aspect of melanization substrate oxidation, since the nonlytic exocytosis experiments were performed in the absence of l-DOPA or other substrates for melanin synthesis.

10.1128/mBio.02085-20.3 FIG S3 Flow cytometry gating strategy. (A and B) H99 and J774.16 cells were prestained with CMFDA and DDAO-SE, respectively, and then coincubated in the presence of IgG1 as an opsonin. After phagocytosis, the cells were washed, stained with Uvitex 2B (which stains extracellular but not phagocytosed fungi), and analyzed by flow cytometry. Plot 1 shows a forward scatter area-versus-side scatter area plot used to gate on macrophage-like cells. Cells in this gate were then plotted on a forward scatter width-versus-forward scatter height graph (plot 2), used for doublet discrimination. Plot 3 shows the gate for J774.16 cells, which are DDAO-SE positive. Finally, a CMFDA-positive, Uvitex 2B-negative gate selects the macrophage-like cells that had internalized C. neoformans. Panel A shows the cells right before sorting, whereas panel B shows plots 3 and 4 for the cells immediately after sorting. (C) The same sample shown in panel B was incubated for an additional 24 h and then analyzed again. This time, the gate in plot 4 was set on CMFDA-negative, DAPI-negative events, which correspond to macrophages that are alive but have no more internalized C. neoformans. Download FIG S3, EPS file, 1.8 MB. Copyright © 2020 Frazão et al. 2020 Frazão et al. This content is distributed under the terms of the Creative Commons Attribution 4.0 International license.

Our results indicate that the frequency of nonlytic exocytosis can differ a great deal depending on the fungal isolate. The discovery that laccase could affect nonlytic exocytosis adds one more melanin-independent role for this crucial enzyme in C. neoformans virulence. Finally, our results corroborate the hypothesis that fungal attributes play a major role in nonlytic exocytosis regulation, as proposed previously (3), and that laccase can be added to the list of fungal attributes involved in this process, such as viability, the capsule, and production of phospholipase B and urease (2, 6, 15).
