# Supplementary figures and images for: Laccase Affects the Rate of Cryptococcus neoformans Nonlytic Exocytosis from Macrophages
Source: mBio. 2020 Sep 8;11(5):e02085-20. doi: 10.1128/mBio.02085-20 (PMC7482070; doi:10.1128/mBio.02085-20)

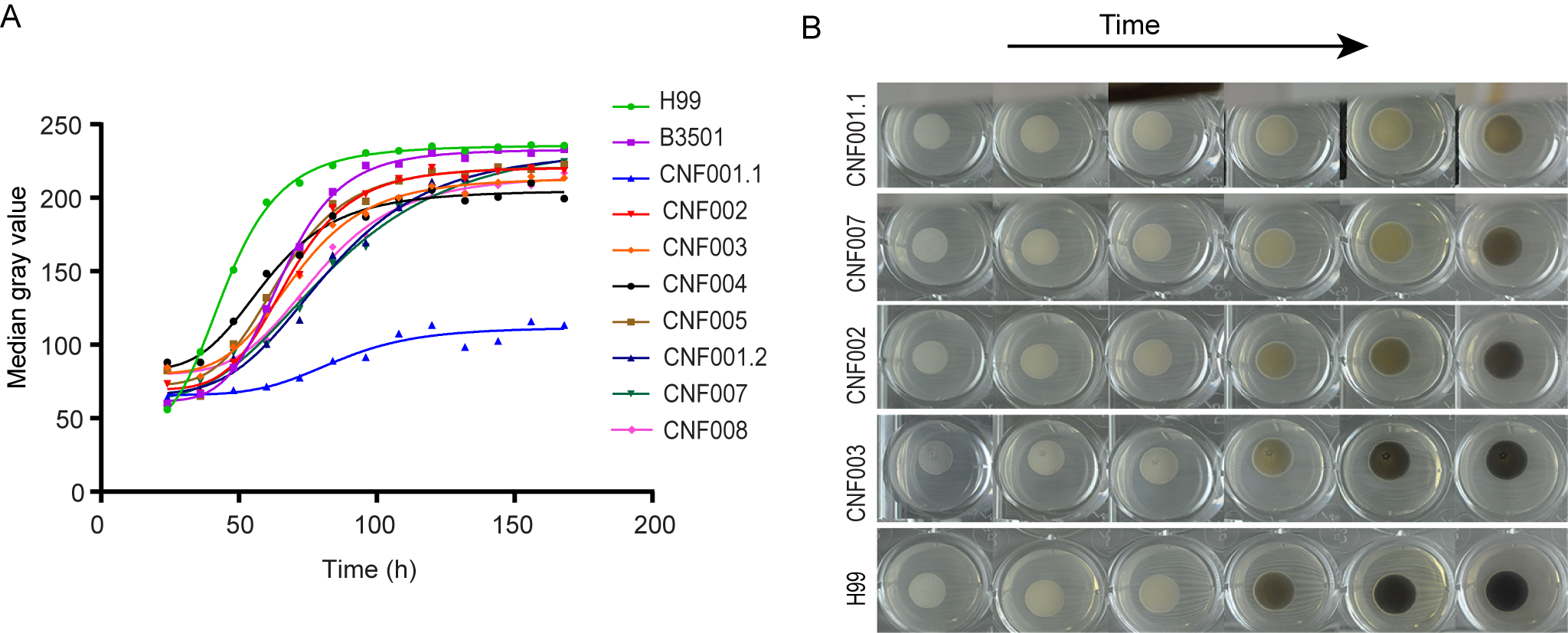

Supplement: FIG S1 [file mBio.02085-20-sf001.tif]
